# Supplementary material for: Hot spot prediction in protein-protein interactions by an ensemble system
Source: BMC Syst Biol. 2018 Dec 31;12(Suppl 9):132. doi: 10.1186/s12918-018-0665-8 (PMC6311905; doi:10.1186/s12918-018-0665-8)
Supplement: Supplementary file 2 — All relevant MATLAB code. A simple Matlab implement of our predictor. (ZIP 19,742 kb) [file 12918_2018_665_MOESM2_ESM.zip › Additional file 2/Read ASA/ASA Property/SKEMPI/2G2UB.txt]

 NetSurfP - Protein Surface Accessibility and Secondary Structure Predictions

  

|  |  |  |
| --- | --- | --- |
|  |  | NetSurfP - Protein Surface Accessibility and Secondary Structure Predictions  Technical University of Denmark |

  


---

```
# For publication of results, please cite:
# A generic method for assignment of reliability scores applied to solvent accessibility predictions.
# Bent Petersen, Thomas Nordahl Petersen, Pernille Andersen, Morten Nielsen and Claus Lundegaard
# BMC Structural Biology 2009, 9:51 doi:10.1186/1472-6807-9-51
#
# Column 1: Class assignment - B for buried or E for Exposed - Threshold: 25% exposure, but not based on RSA
# Column 2: Amino acid
# Column 3: Sequence name
# Column 4: Amino acid number
# Column 5: Relative Surface Accessibility - RSA
# Column 6: Absolute Surface Accessibility
# Column 7: Z-fit score for RSA prediction
# Column 8: Probability for Alpha-Helix
# Column 9: Probability for Beta-strand
# Column 10: Probability for Coil
E A  2G2UB                  1    0.833  91.753  -0.289   0.003   0.003   0.994
E G  2G2UB                  2    0.564  44.418  -2.041   0.019   0.141   0.840
E V  2G2UB                  3    0.469  72.039  -1.083   0.019   0.141   0.840
B M  2G2UB                  4    0.199  39.900  -1.305   0.056   0.142   0.802
E T  2G2UB                  5    0.463  64.163   0.582   0.113   0.087   0.800
E G  2G2UB                  6    0.364  28.655  -0.777   0.354   0.048   0.598
E A  2G2UB                  7    0.656  72.258  -0.136   0.278   0.093   0.628
E K  2G2UB                  8    0.438  90.117   0.792   0.307   0.165   0.527
B F  2G2UB                  9    0.138  27.656   0.360   0.321   0.252   0.427
E T  2G2UB                 10    0.483  66.937  -0.114   0.231   0.330   0.439
E Q  2G2UB                 11    0.430  76.798   0.519   0.252   0.423   0.325
B I  2G2UB                 12    0.096  17.834   0.105   0.159   0.516   0.325
E Q  2G2UB                 13    0.348  62.206  -0.100   0.159   0.516   0.325
B F  2G2UB                 14    0.187  37.551  -0.427   0.159   0.516   0.325
E G  2G2UB                 15    0.262  20.627  -0.951   0.135   0.317   0.548
B M  2G2UB                 16    0.183  36.538  -0.097   0.199   0.152   0.649
E T  2G2UB                 17    0.342  47.394  -0.163   0.307   0.165   0.527
B R  2G2UB                 18    0.176  40.396   0.398   0.701   0.107   0.192
E Q  2G2UB                 19    0.503  89.782   0.100   0.701   0.107   0.192
B Q  2G2UB                 20    0.243  43.364   1.191   0.649   0.163   0.188
B V  2G2UB                 21    0.048   7.378   0.511   0.662   0.225   0.113
B L  2G2UB                 22    0.132  24.242   1.062   0.662   0.225   0.113
E D  2G2UB                 23    0.467  67.237   0.545   0.662   0.225   0.113
B I  2G2UB                 24    0.077  14.208  -0.199   0.502   0.102   0.396
B A  2G2UB                 25    0.184  20.233  -0.593   0.386   0.097   0.517
E G  2G2UB                 26    0.402  31.614  -1.045   0.113   0.087   0.800
B A  2G2UB                 27    0.246  27.087  -1.333   0.052   0.084   0.864
E E  2G2UB                 28    0.567  99.125  -0.428   0.056   0.142   0.802
E N  2G2UB                 29    0.436  63.801  -0.376   0.056   0.142   0.802
B C  2G2UB                 30    0.167  23.405  -0.741   0.056   0.142   0.802
E E  2G2UB                 31    0.570  99.579  -0.548   0.118   0.150   0.732
E T  2G2UB                 32    0.484  67.117  -0.744   0.113   0.087   0.800
E G  2G2UB                 33    0.348  27.395  -1.666   0.115   0.016   0.868
E G  2G2UB                 34    0.354  27.860  -1.394   0.053   0.043   0.903
E S  2G2UB                 35    0.443  51.884  -1.101   0.056   0.142   0.802
E F  2G2UB                 36    0.296  59.508  -0.170   0.069   0.386   0.545
B G  2G2UB                 37    0.227  17.881  -1.120   0.021   0.451   0.528
E D  2G2UB                 38    0.391  56.401  -0.964   0.021   0.451   0.528
B S  2G2UB                 39    0.207  24.249   0.030   0.021   0.756   0.223
B I  2G2UB                 40    0.035   6.512  -0.201   0.018   0.846   0.136
B H  2G2UB                 41    0.092  16.789   0.709   0.011   0.918   0.071
B C  2G2UB                 42    0.020   2.752   0.502   0.018   0.846   0.136
B R  2G2UB                 43    0.139  31.785   0.509   0.023   0.655   0.322
B G  2G2UB                 44    0.241  19.006  -1.693   0.005   0.336   0.660
B H  2G2UB                 45    0.286  52.078   0.198   0.019   0.141   0.840
E A  2G2UB                 46    0.434  47.816  -1.600   0.018   0.088   0.893
E A  2G2UB                 47    0.490  53.954  -1.383   0.053   0.043   0.903
E G  2G2UB                 48    0.420  33.046  -1.796   0.053   0.043   0.903
E D  2G2UB                 49    0.410  59.067  -1.148   0.052   0.084   0.864
B Y  2G2UB                 50    0.244  52.121  -0.935   0.056   0.142   0.802
B Y  2G2UB                 51    0.201  43.039  -0.404   0.056   0.142   0.802
B A  2G2UB                 52    0.187  20.596  -0.430   0.018   0.088   0.893
B Y  2G2UB                 53    0.183  39.043  -0.808   0.019   0.141   0.840
E A  2G2UB                 54    0.264  29.060  -1.053   0.019   0.141   0.840
B T  2G2UB                 55    0.150  20.819  -2.038   0.066   0.296   0.638
B F  2G2UB                 56    0.090  18.103  -0.337   0.069   0.386   0.545
B G  2G2UB                 57    0.117   9.176  -1.163   0.074   0.484   0.442
B F  2G2UB                 58    0.069  13.868  -0.413   0.069   0.386   0.545
B T  2G2UB                 59    0.151  20.971  -0.557   0.066   0.296   0.638
E S  2G2UB                 60    0.349  40.903  -0.907   0.064   0.216   0.721
B A  2G2UB                 61    0.254  27.958  -1.016   0.199   0.152   0.649
E A  2G2UB                 62    0.327  36.068  -1.309   0.191   0.086   0.723
E A  2G2UB                 63    0.430  47.364  -0.930   0.278   0.093   0.628
E D  2G2UB                 64    0.524  75.523  -0.490   0.278   0.093   0.628
E A  2G2UB                 65    0.308  33.975  -0.818   0.278   0.093   0.628
E K  2G2UB                 66    0.497 102.274   0.497   0.307   0.165   0.527
B V  2G2UB                 67    0.146  22.440  -0.089   0.321   0.252   0.427
B D  2G2UB                 68    0.212  30.564   0.320   0.321   0.252   0.427
E S  2G2UB                 69    0.398  46.634  -0.526   0.453   0.248   0.299
E K  2G2UB                 70    0.508 104.434   0.349   0.502   0.102   0.396
E S  2G2UB                 71    0.400  46.903  -0.151   0.502   0.102   0.396
E Q  2G2UB                 72    0.359  64.100  -0.132   0.502   0.102   0.396
E E  2G2UB                 73    0.459  80.100  -0.215   0.502   0.102   0.396
E K  2G2UB                 74    0.485  99.867   0.369   0.386   0.097   0.517
B L  2G2UB                 75    0.160  29.296  -0.785   0.278   0.093   0.628
B L  2G2UB                 76    0.172  31.548   0.222   0.184   0.043   0.773
E A  2G2UB                 77    0.530  58.384   0.072   0.113   0.043   0.844
E P  2G2UB                 78    0.303  43.052  -0.553   0.058   0.017   0.925
E S  2G2UB                 79    0.681  79.766  -1.085   0.018   0.019   0.964
E A  2G2UB                 80    0.489  53.855  -0.330   0.018   0.019   0.964
B P  2G2UB                 81    0.259  36.695  -0.851   0.018   0.019   0.964
E T  2G2UB                 82    0.399  55.286  -0.462   0.064   0.216   0.721
B L  2G2UB                 83    0.158  28.948  -0.195   0.079   0.592   0.329
E T  2G2UB                 84    0.382  52.942   0.738   0.087   0.683   0.230
B L  2G2UB                 85    0.130  23.876   0.035   0.079   0.592   0.329
B A  2G2UB                 86    0.234  25.798  -0.819   0.159   0.516   0.325
E K  2G2UB                 87    0.415  85.283   0.161   0.159   0.516   0.325
B F  2G2UB                 88    0.183  36.708   0.075   0.148   0.418   0.435
E N  2G2UB                 89    0.410  60.068  -0.858   0.135   0.317   0.548
E Q  2G2UB                 90    0.431  76.941   0.071   0.231   0.330   0.439
B V  2G2UB                 91    0.264  40.546  -1.145   0.231   0.330   0.439
E T  2G2UB                 92    0.350  48.476  -0.412   0.231   0.330   0.439
B V  2G2UB                 93    0.246  37.779  -0.352   0.231   0.330   0.439
B G  2G2UB                 94    0.222  17.464  -1.558   0.321   0.252   0.427
B M  2G2UB                 95    0.171  34.277   0.004   0.428   0.171   0.402
B T  2G2UB                 96    0.232  32.192  -0.507   0.453   0.248   0.299
E R  2G2UB                 97    0.296  67.807  -0.004   0.578   0.229   0.194
B A  2G2UB                 98    0.147  16.254  -0.467   0.578   0.229   0.194
E Q  2G2UB                 99    0.334  59.742   0.286   0.594   0.285   0.121
B V  2G2UB                100    0.092  14.187   0.022   0.487   0.370   0.144
B L  2G2UB                101    0.123  22.503   0.317   0.478   0.309   0.214
B A  2G2UB                102    0.236  25.963  -0.823   0.478   0.309   0.214
B T  2G2UB                103    0.244  33.871  -0.927   0.453   0.248   0.299
B V  2G2UB                104    0.104  16.016   0.657   0.199   0.152   0.649
E G  2G2UB                105    0.340  26.774  -0.627   0.018   0.047   0.935
E Q  2G2UB                106    0.592 105.678  -0.262   0.018   0.019   0.964
E G  2G2UB                107    0.562  44.214  -2.228   0.053   0.043   0.903
E S  2G2UB                108    0.346  40.516  -0.549   0.113   0.087   0.800
B C  2G2UB                109    0.075  10.502  -0.948   0.135   0.317   0.548
E T  2G2UB                110    0.318  44.065  -0.085   0.135   0.317   0.548
E T  2G2UB                111    0.390  54.038  -0.885   0.216   0.235   0.548
B W  2G2UB                112    0.260  62.434   0.199   0.428   0.171   0.402
B S  2G2UB                113    0.298  34.972  -1.637   0.428   0.171   0.402
E E  2G2UB                114    0.506  88.346  -0.924   0.307   0.165   0.527
E Y  2G2UB                115    0.444  94.840  -0.643   0.199   0.152   0.649
B Y  2G2UB                116    0.175  37.483   0.624   0.052   0.084   0.864
E P  2G2UB                117    0.418  59.272  -0.875   0.053   0.043   0.903
E A  2G2UB                118    0.546  60.202  -1.587   0.018   0.047   0.935
B Y  2G2UB                119    0.183  39.086   0.510   0.018   0.047   0.935
E P  2G2UB                120    0.369  52.304  -0.709   0.052   0.084   0.864
B S  2G2UB                121    0.291  34.058  -1.153   0.113   0.043   0.844
E T  2G2UB                122    0.576  79.947  -0.742   0.113   0.043   0.844
E A  2G2UB                123    0.441  48.642  -0.647   0.113   0.043   0.844
E G  2G2UB                124    0.399  31.378  -1.799   0.052   0.084   0.864
B V  2G2UB                125    0.269  41.391  -0.617   0.066   0.296   0.638
E T  2G2UB                126    0.416  57.630  -0.436   0.023   0.655   0.322
E L  2G2UB                127    0.300  54.875   0.157   0.021   0.756   0.223
E S  2G2UB                128    0.407  47.724  -0.020   0.021   0.756   0.223
B L  2G2UB                129    0.097  17.834   0.430   0.021   0.756   0.223
B S  2G2UB                130    0.177  20.803  -0.503   0.018   0.846   0.136
B C  2G2UB                131    0.032   4.535  -1.695   0.021   0.756   0.223
B F  2G2UB                132    0.241  48.449  -0.829   0.022   0.552   0.426
E D  2G2UB                133    0.395  56.977  -1.034   0.022   0.359   0.619
E V  2G2UB                134    0.315  48.431  -0.994   0.020   0.205   0.775
E D  2G2UB                135    0.649  93.579  -1.941   0.018   0.088   0.893
E G  2G2UB                136    0.488  38.390  -1.186   0.053   0.043   0.903
B Y  2G2UB                137    0.284  60.669  -1.335   0.053   0.043   0.903
E S  2G2UB                138    0.389  45.626  -1.080   0.052   0.084   0.864
E S  2G2UB                139    0.479  56.174  -0.237   0.056   0.142   0.802
E T  2G2UB                140    0.380  52.706  -1.102   0.056   0.142   0.802
E G  2G2UB                141    0.402  31.637  -1.713   0.020   0.205   0.775
E F  2G2UB                142    0.316  63.381  -0.896   0.021   0.279   0.699
B Y  2G2UB                143    0.136  29.063  -0.249   0.021   0.279   0.699
E R  2G2UB                144    0.382  87.386  -0.060   0.018   0.088   0.893
E G  2G2UB                145    0.330  25.979  -2.218   0.018   0.088   0.893
B S  2G2UB                146    0.272  31.890  -1.202   0.020   0.205   0.775
B A  2G2UB                147    0.102  11.273  -1.598   0.021   0.451   0.528
B H  2G2UB                148    0.233  42.455  -0.187   0.021   0.756   0.223
B L  2G2UB                149    0.054   9.869   0.375   0.018   0.846   0.136
B W  2G2UB                150    0.184  44.204   0.384   0.011   0.918   0.071
B F  2G2UB                151    0.071  14.350  -0.199   0.021   0.756   0.223
E T  2G2UB                152    0.319  44.218  -0.272   0.023   0.655   0.322
E D  2G2UB                153    0.454  65.465  -1.151   0.021   0.279   0.699
B G  2G2UB                154    0.283  22.248  -1.452   0.022   0.359   0.619
E V  2G2UB                155    0.357  54.932   0.355   0.023   0.655   0.322
B L  2G2UB                156    0.162  29.681  -0.468   0.021   0.756   0.223
E Q  2G2UB                157    0.406  72.565  -0.213   0.023   0.655   0.322
B G  2G2UB                158    0.311  24.468  -1.232   0.079   0.592   0.329
E K  2G2UB                159    0.462  95.033  -0.103   0.085   0.775   0.140
E R  2G2UB                160    0.396  90.592  -0.667   0.087   0.683   0.230
E Q  2G2UB                161    0.410  73.226  -0.833   0.074   0.484   0.442
E W  2G2UB                162    0.397  95.527  -1.098   0.069   0.386   0.545
E D  2G2UB                163    0.613  88.261  -0.625   0.118   0.150   0.732
E L  2G2UB                164    0.329  60.185  -0.694   0.113   0.087   0.800
E V  2G2UB                165    0.778 119.532  -1.147   0.003   0.003   0.994

Explain the output.  Go back.
```

---
